# The effect of anti-rheumatic medications for coronary artery diseases risk in patients with rheumatoid arthritis might be changed over time: A nationwide population-based cohort study

**DOI:** 10.1371/journal.pone.0179081

**Published:** 2017-06-28

**Authors:** Yao-Min Hung, Lichi Lin, Chyong-Mei Chen, Jeng-Yuan Chiou, Yu-Hsun Wang, Paul Yung-Pou Wang, James Cheng-Chung Wei

**Affiliations:** 1 Department of Emergency Medicine, Kaohsiung Veterans General Hospital, Kaohsiung, Taiwan; 2 Institute of Public Health, School of Medicine, National Yang Ming University, Taipei, Taiwan; 3 Yuhing Junior College of Health Care and Management, Kaohsiung, Taiwan; 4 Department of Statistics, Oklahoma state University, Stillwater, OK, United States of America; 5 Division of Allergy, Immunology and Rheumatology, Chung Shan Medical University Hospital, and Institute of Medicine, Chung Shan Medical University, Taichung, Taiwan; 6 School of Health Policy and Management, Chung Shan Medical University, Taichung, Taiwan; 7 Department of Medical Research, Chung Shan Medical University, Taichung, Taiwan; 8 Division of Nephrology, Kaiser Permanente Baldwin Park Medical Center, Baldwin Park, CA, United States of America; 9 Graduate Institute of Integrated Medicine, China Medical University, Taichung, Taiwan; University of Birmingham, UNITED KINGDOM

## Abstract

**Objectives:**

To determine whether anti-rheumatic drug usage is associated with risk of coronary artery diseases (CAD) in incident Rheumatoid Arthritis (RA) patients.

**Methods:**

Data were obtained from the Taiwan National Health Insurance Research Database. The study cohort comprised 6260 patients who were newly diagnosed with RA between 2001–2010. The study endpoint was occurrence of CAD according to the ICD-9-CM codes. We used the WHO Defined Daily Dose (DDD) as a tool to assess the drugs exposure. The Cox proportional hazards regression model was used to estimate the hazard ratio (HR) of disease after controlling for demographic and other co-morbidities. When the proportionality assumption is violated, a spline curve of the Scaled Schoenfeld residuals is fitted to demonstrate the estimated effect on CAD over time for drug usage.

**Results:**

Among RA patients, use of celecoxib, and etoricoxib was associated with significantly decreased incidence of CAD. The adjusted HR(95% CI) of CAD for low-dose celecoxib (DDD≦1) and high-dose user were 0.47(0.34, 0.65) and 0.37(0.24, 0.58) during the 4 year follow-up time; however, it became 0.98(0.70, 1.37) and1.29(0.85, 1.95). Adjusted HR(95% CI) of CAD for etoricoxib users remained 0.47(0.26, 0.84).

**Conclusions:**

This study revealed association of decreased CAD risk in RA patients taking 2 different kinds of COX-2i in comparison with nonusers. The effect might be changed over time, after about 4 years.

## Introduction

Rheumatoid arthritis (RA) is a common and destructive chronic systemic inflammatory disease with the average age-adjusted annual incidence rate 15.8 per 100,000 in Taiwan[1,2]. Although joint involvement is the prototypical pathology of RA, atherosclerotic cardiovascular disease is the major cause of mortality and morbidity of RA in many studies [3–7]. A systematic review and meta-analysis has shown that there is an increased incidence of cardiovascular events in patients with RA, such as acute myocardial infarction, stroke, and cardiac death[3]. Most studies investigating mortality in patients with RA compared to those without RA have found increased mortality rates largely due to cardiovascular diseases(CVD), specifically coronary artery diseases(CAD) [8–10]. Previous meta-analysis which included 24 observational studies have shown increased risk of mortality from CAD was increased by 59% among patients with RA[8].

Kaplan MJ claimed that the lack of a unifying explanation for accelerated CVD in RA is reflected by the confusion that still exists regarding possible preventive measures aimed at decreasing atherogenic risk[11]. Over the past few years, there has been mounting evidence of a series of factors related to inflammation which explain accelerated CVD in RA[12,13].Both traditional Framingham risk factors and such risk factors unique to those with systemic inflammatory disorders may have contributed to CAD risk in RA patients[6,14–16]. To clarify this phenomenon, some postulated that anti-rheumatic medications appear to modify the risk of CVD in RA[17]. Among anti-rheumatic medications, disease-modifying anti-rheumatic drugs (DMARDs), such as sulfasalazine, hydroxychloroquine or methotrexate, have been associated with reduced risk or mortality of CVD in RA patients in several studies[17–21]. Anti–tumor necrosis factor (anti-TNF) agents (for example, etanercept) may decrease the risk of new cardiovascular events in some observational studies[22–23]. Cyclooxygenase-2 inhibitors (such as rofecoxib and valdecoxib) usage has been associated with an increased risk of CVD in the general population in some studies[24–28], while others did not show evidence of increased risk for celecoxib[29–30]. A case-control analysis within a cohort of subjects with RA, observed between 1999 and 2003 showed that risk of acute myocardial infarction was not related with cyclooxygenase 2 inhibitors (COX-2i) usage (rate ratio 1.11, 95% confidence interval 0.87–1.43) [20].

There remains considerable uncertainty about the effect of COX-2i on the risk of CAD among RA patients, because these medications might have dual effects on risk for CV morbidity[11]. Some studies showed the use of rofecoxib or celecoxib was associated with an increased cardiovascular risk among patients with a history of colorectal adenomas [25,26]. COX-2i suppress vascular production of prostacyclin without affecting the synthesis of platelet-derived thromboxane A_2_, which may promote thrombosis and increase the risk of cardiovascular events [31]. However, COX-2i have an ability to reduce systemic inflammation, which might decrease CV risk. Some population-based studies found that the use of celecoxib had negative association with cardiovascular diseases in patients with ankylosing spondylitis [32,33]. As previous studies about this issue were conducted in western countries and most of them have not evaluated the role of time-varying drug effect in study designs, we aim to conduct a nationwide population-based cohort study to determine such effects. The objective of this study is to evaluate the possible effects of RA drugs on CAD and to see whether time-varying drug effect exist in Asia incident RA patients. The Taiwanese National Health Insurance Research Database (NHIRD) is used to solve this problem.

## Materials and methods

### Study design and data sources

The data sources used for this study was the claims data of Taiwan’s National Health Insurance Research Database (NHIRD), which contains health care data of virtually all residents in Taiwan. Taiwan’s NHIRD is a publically-released and de-identified research database that virtually covers 99.6% of 2.3 million population in Taiwan. Within Taiwan’s national health insurance (NHI) scheme, medical claims are sent to the Bureau of National Health Insurance (BNHI) of Taiwan for cross-checking and validation with the aim of ensuring the accuracy of diagnosis coding.

In this cohort study, we used a subset of NHIRD, the Longitudinal Health Insurance Database (LHID), which comprises the patient data from 2000 to 2010. The LHID includes the original claim data of 1,000,000 beneficiaries randomly sampled from the original NHIRD. The database includes encrypted personal information, such as demographic characteristics, disease diagnoses, and prescription records (including types of medication, time of prescription, duration of drug supply, and dosage).The diagnosis of disease is based on the International Classification of Diseases, Ninth Revision, Clinical Modification (ICD-9-CM codes). There were no statistically significant differences of distribution in age, gender, or health care costs between the 1,000,000 patients from the LHID database and the original NHIRD, as reported by the National Health Research Institute of Taiwan. This study was approved by the Institutional Review Board of Chung Shan Medical University in Taiwan (CSMUH CS15134). As data used consisted of deidentified secondary data set released for research purposes and were analyzed anonymously, the need for informed consent was waived, which comply with the regulations of the Department of Health, Taiwan.

### Study sample

Our study identified newly diagnosed patients with RA between 2001 and 2009 from both ambulatory and inpatient care as the study cohort (ICD-9-CM code 714.0). The index visit was defined as the first medical visit, during which the diagnosis of RA was made. To ensure only patients with correct diagnosis of RA was included, only patients with at least one inpatient admission or three new ambulatory visits (including the index visit) with a diagnosis of RA between January 1, 2001 and December 31, 2009 were considered for inclusion in the RA group. The exclusion criteria for the recruitment of subjects into the RA group were: (1) age less than 18 years; (2) a previous diagnosis of RA during year 1999–2000 to increase the likelihood of identifying only newly diagnosed RA cases in 2001; (3) a previous diagnosis of CAD (ICD-9-CM codes 410–414) before the index visit. As a result, a total of 6260 CAD-free subjects with newly diagnosed RA study cohort were identified from the LHID 2000 for the study based on the above criteria. The age of each study subject was measured by the difference in time between the index date and the date of birth.

### Outcome and follow-up

All the ambulatory medical care and inpatient records for each subject in the two groups were tracked from their index visit till the end of 2010. The date of the first principal diagnosis of CAD during the follow-up period was defined as the primary endpoint. The diagnosis of CAD definition in this study is based on the ICD-9-CM codes 410–414. 410 means “Acute myocardial infarction”, 411 means “Other acute and subacute forms of ischaemic heart disease”, 412 means “Old myocardial infarction”, 413 means “Angina pectoris “, 414 means “Other forms of chronic ischemic heart disease”. All subjects were followed from the index visit to the first occurrence of CAD, withdraw from the national health insurance or end of follow-up.

First, we evaluated the effect of RA on CAD-free survival, adjusting for demographic features (age and sex) along with the preexisting cardiovascular comorbidities of hypertension (ICD-9-CM code 401–405), diabetes (ICD-9-CM code 250), hyperlipidemia (ICD-9-CM code 272) and Charlson comorbidity index. Information on comorbid medical disorders was obtained by tracing all the ambulatory medical care and inpatients records in the NHI database in the year before the index visit. All study subjects were followed-up until December 31, 2010, death or record any incident CAD which ever event came first.

Subsequently, we evaluated the effect of different RA drugs on CAD in the RA study cohort. The drug use was defined as usage of each listed drugs > 90 days after diagnosis of Rheumatoid Arthritis. If the duration of listed drugs less than 90 days, we just define as non-users. We calculated the total cumulated dose of each listed drugs from the index date of newly diagnosed RA until the first occurrence of CAD or the end of follow up. We used the World Health Organization defined daily dose (DDD) as a tool to assess the frequency of drugs exposure. DDD is defined as the assumed average maintenance dose per day for a drug used for its main indication in adults.[34] According to DDD, patients taking celecoxib were divided into 2 groups: ≦ 1 DDD, > 1 DDD(1 DDD = 200 mg/day); patients taking methotrexate were divided into two groups: ≦ 0.5 DDD and >0.5 DDD(1 DDD = 2.5 mg/day); patients taking hydroxychloroquine were divided into two groups: ≦0.5 DDD and > 0.5 DDD(1 DDD = 516 mg/day). patients taking sulfasalazine were not divided. The low-dose was defined as ≦ 1 DDD and high-dose groups as > 1 DDD (200 gm per day for celebrex and 60 mg per day for etoricoxib). Risk factors of CAD including sex, age, Charlson comorbidity index(CCI), RA disease duration, diabetes (DM), hypertension, hyperlipidemia were considered as potential confounding factors and incorporated into the models.

### Statistical analysis

Cox proportional hazard regressions were used to assess the risk associated with CAD. Hazard ratio (HR) and 95% confidence interval (CI) were calculated in the model. Furthermore, we evaluated the effect of COX-2i on CAD on RA study cohort.

Cox proportional hazard regression was used to assess how the different drugs and doses affected the risk of incident CAD in subjects with RA. The Cox proportional hazards regression model was used to estimate the hazard ratio (HR) of disease after controlling for demographic and other co-morbidities. The Chi-square test is used to test for non-proportionality of hazards in the Cox model. When the proportionality assumption is violated, aspline curve of the Scaled Schoenfeld residuals is fitted to demonstrate the estimated preventive effect on CAD over time for drug usage [35]. The statistical analyses in this study were executed by R software version 3.2.0 (2015-04-16) [36] and the significance level was set to be 0.05. The Cox proportional hazard analysis was also used to estimate whether there were reduced CAD risks associated with antirheumatic medications.

## Results

Our study included 6260 incident RA patients. Table 1 showed distributions of demographic characteristics, comorbidities and antirheumatic drug use among the RA cohort. The median and range of followup times is 5.39 year and 0.25 to 10 years Table 2 showed the Cox proportional Hazard model for RA patients- baseline covariate effects (demographic characteristics, comorbidities and antirheumatic drug use) of developing CAD. During the 10 -year follow-up period from the index date, 20.1% (1253 /6260) of incident RA patients developed CAD. The crude HR of CAD for males was 1.25((95% CI, 1.11, 1.40)compared with females. Patients having the comorbidities of diabetes, hypertension, hyperlipidemia have increased risk of developing CAD. Comparing with the patients having CCI score 0, crude HR of CAD for the group of CCI score 1 was 1.24(95% CI,0.71, 2.13), whereas crude HR of CAD for the group of CCI score 2 was 1.87(95% CI,1.06, 3.30; p<0.05). Table 3 showed the demographic and baseline differences for comparing drug users and nonusers for RA patients.

**Table 1:** Demographic characteristics comorbidities and anti-rheumatic drug use among 6260 incident RA patients.

	Rheumatoid arthritis (N = 6260)	
	n	%
**Gender**		
Female	4394	70.2
Male	1866	29.8
**Age, mean±SD**	53.8 ± 13.2	
**CCI**^**a**^		
0	84	1.3
1	5611	89.6
≧2	565	9.0
**Hypertension**	1477	23.6
**Hyperlipidemia**	872	13.9
**Celebrex**	936	15.0
**Etoritoxib**	156	2.5
**Hydroxychloroquine**	962	15.4
**Methotrexate**	571	9.1
**Etanercept **	56	0.9
**Sulfasalazine**	541	8.6

^a^ Charlson comorbidity index.

**Table 2:** Cox proportional Hazard model for RA patients-baseline covariate effects.

	No. of patients (N = 6260)	No. (%) of CAD events (Ne = 1253)	Log-rank test (Mantel–Cox test) *p*-value	Crude HR and 95% CI
**Gender**				
female	4394	817	0.0002	1
male	1866	436		1.25 (1.11–1.40)^2^
**Age of diagnosis of RA, mean±SD**	53.8 ± 13.2			
≥18 and ≤40	864	61		1
>40 and ≤60	3471	586	0	2.71 (2.09–3.53)^2^
>60	1925	606		5.81 (4.46–7.56)^2^
**CCI**^**a**^				
0	84	13	5.87e-06	1
1	5611	1089		1.24 (0.71–2.13)
≥2	565	151		1.87 (1.06, 3.30)^1^
**Hypertension**				
no	4783	785	0	1
yes	1477	468		2.27 (2.03–2.55)^2^
**Hyperlipidemia**				
no	5388	1031	1.88e-08	1
yes	872	222		1.51 (1.31–1.75)^2^

CAD, coronary artery disease; CI, confidence interval; HR, Hazard ratios.

^a^ Charlson comorbidity index.

^1^ p<0.05

^2^ p<0.001.

**Table 3:** Demographic and baseline differences for comparing drug users and nonusers for RA patients.

	Celecoxib			Etoricoxib			MTX			HCQ			SSZ			
	No	Yes		No	Yes		No	Yes		No	Yes		No	Yes		*Total*
	(N = 5324)	(N = 936)	*p-value*	(N = 6104)	(N = 156)	*p-value*	(N = 5689)	(N = 571)	*p-value*	(N = 5298)	(N = 962)	*p-value*	(N = 5719)	(N = 541)	*p-value*	*(N = 6260)*
**Age**			*p<0*.*0001*			*p = 0*.*0563*			*p<0*.*0001*			*p = 0*.*0004*			*p = 0*.*0002*	
18–40	762(14.3)	102(10.9)		848(13.9)	16(10.3)		769(13.5)	95(16.6)		707(13.3)	157(16.3)		767(13.4)	97(17.9)		864
40–60	3038(57.1)	433(46.3)		3392(55.6)	79(50.6)		3120(54.8)	351(61.5)		2914(55.0)	557(57.9)		3157(55.2)	314(58.0)		3471
>60	1524(28.6)	401(42.8)		1864(30.5)	61(39.1)		1800(31.6)	125(21.9)		1677(31.7)	248(25.8)		1795(31.4)	130(24.0)		1925
**Gender**			*p = 0*.*0075*			*p = 0*.*0026*			*p = 0*.*0004*			*p<0*.*0001*			*p = 0*.*2474*	
Female	3702(69.5)	692(73.9)		4267(69.9)	127(81.4)		3956(69.5)	438(76.7)		3645(68.8)	749(77.9)		4002(70)	392(72.5)		4394
Male	1622(30.5)	244(26.1)		1827(30.1)	29(18.6)		1733(30.5)	133(23.3)		1653(31.2)	213(22.1)		1717(30)	149(27.5)		1866
**CCI**			*p = 0*.*0085*			*p = 0*.*7835*			*p = 0*.*0243*			*p = 0*.*0259*			*p = 0*.*0139*	
0	65(1.2)	19(2.0)		81(1.3)	3(1.9)		72(1.3)	12(2.1)		63(1.2)	21(2.2)		72(1.3)	12(2.2)		84
1	4797(90.1)	814(87.0)		5471(89.6)	140(89.7)		5089(89.5)	522(91.4)		4747(89.6)	864(89.8)		5116(89.5)	495(91.5)		5611
> = 2	462(8.7)	103(11.0)		552(9.0)	13(8.3)		528(9.3)	37(6.5)		488(9.2)	77(8.0)		531(9.3)	34(6.3)		565
**Hypertension**			*p = 0*.*0133*			*p = 0*.*6595*			*p<0*.*0001*			*p = 0*.*0005*			*p = 0*.*0007*	
No	4098(77)	685(73.2)		4661(76.4)	122(78.2)		4308(75.7)	475(83.2)		4005(75.6)	778(80.9)		4337(75.8)	446(82.4)		4783
yes	1226(23)	251(26.8)		1443(23.6)	34(21.8)		1381(24.3)	96(16.8)		1293(24.4)	184(19.1)		1382(24.2)	95(17.6)		1477
**Hyperlipidemia**			*p = 0*.*099*			*p = 0*.*1129*			*p = 0*.*0566*			*p = 0*.*0484*			*p = 0*.*003*	
No	4599(86.4)	789(84.3)		5261(86.2)	127(81.4)		4881(85.8)	507(88.8)		4540(85.7)	848(88.1)		4899(85.7)	489(90.4)		5388
Yes	725(13.6)	147(15.7)		843(13.8)	29(18.6)		808(14.2)	64(11.2)		758(14.3)	114(11.9)		820(14.3)	52(9.6)		762

Number and percentage in parentheses for each category are shown and the Pearson’s chi-squared test (with Yates' continuity correction) is used to assess the difference of the baseline distributions.

### COX-2 inhibitors Might Reduce CAD in the RA cohort from single drug effect by DDD

**Fig 1:**
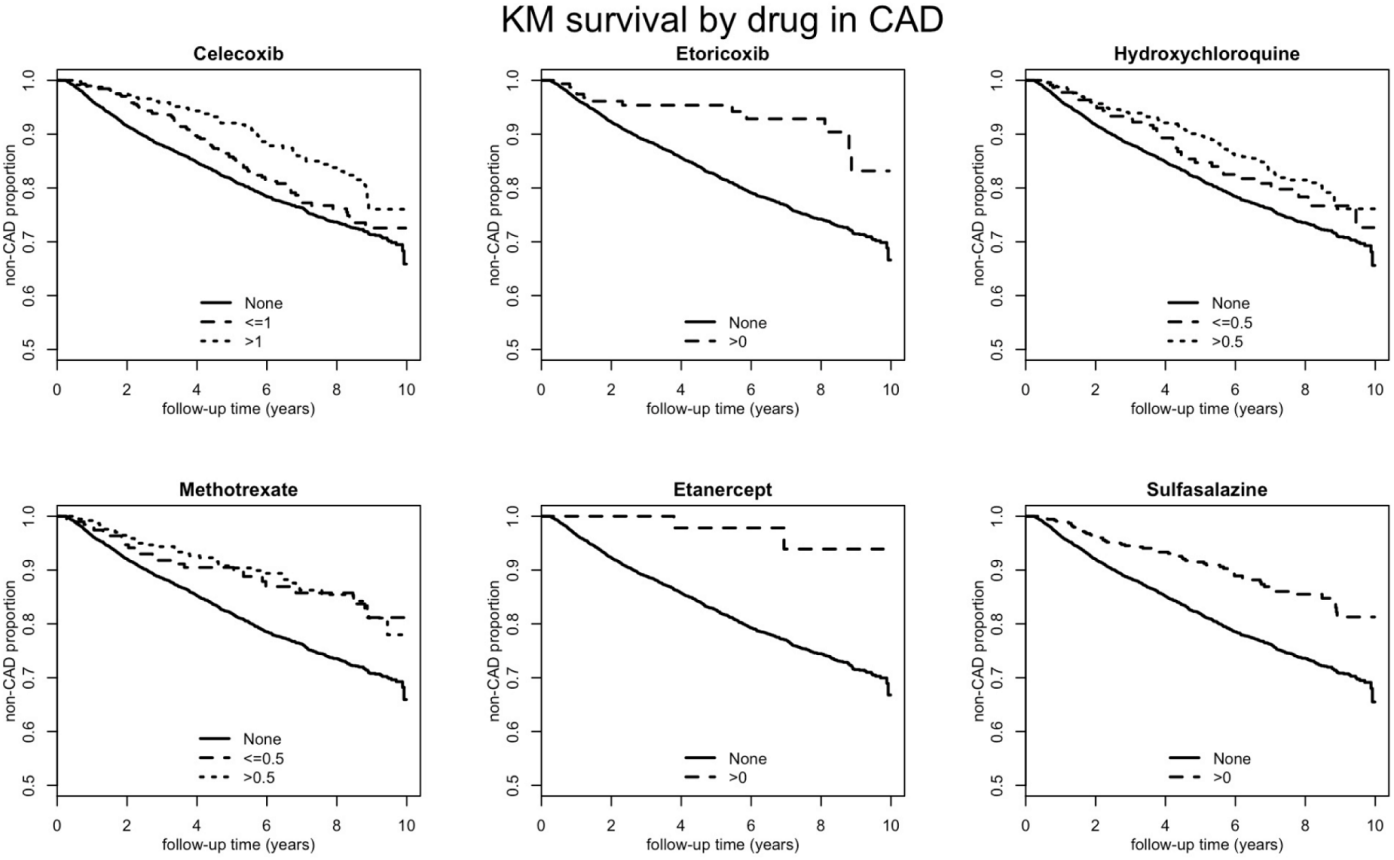
Kaplan Meier survival curves by single drug in CAD. Celebrex 1 DDD = 200 mg; Etoritoxib 1 DDD = 60 mg; Hydroxychloroquine 1 DDD = 516 mg; Methotrexate 1 DDD = 2.5 mg; Etanercept 1 DDD = 7 mg; Sulfasalazine 1 DDD = 2000 mg.

Table 4 shows the effect of antirheumatic drug use on CAD in the RA study cohort by different DDD status of each drug. The crude and adjusted HR of CAD in the RA study cohort by each drug were shown. Among RA patients, COX-2i use was associated with significantly decreased incidence of CAD. This phenomenon was observed among 2 types of COX-2i, celecoxib, and etoricoxib. Analyzing the defined daily dose of COX-2i indicated that both low- and high-dose groups exhibited significantly decreased CAD events compared with celecoxib nonusers. The Kaplan Meier curves were demonstrated to reveal initially the covariate and drug differences in predicting and comparing risks of CAD. Fig 1 showed Kaplan Meier survival curves by single drug in CAD.

**Table 4:** Cox proportional hazard model for RA patients–single drug effect.

	No. of patients (N = 6260)	No. (%) of CAD events (Ne = 1253)	Log-rank test (Mantel–Cox test) *p*-value	Crude (Unadjusted) HR and 95% CI	Adjusted HR and 95% CI	*p*-value for testing proportionality of Cox model	
						Unadjusted	Adjusted^a^
**Celecoxib**							
None	5324	1109	9.28e-06	ref	ref	ref	ref
≤1	451	83		0.82(0.65, 1.02)^1^	0.63(0.50, 0.79)^4^	1.04e-03	3.29e-04
>1	485	61		0.55(0.43, 0.72)^4^	0.63(0.47, 0.85)^3^	7.03e-07	1.34e-05
**Etoricoxib**							
None	6104	1241	0.0004	Ref	ref	ref	ref
>0	156	12		0.37(0.21, 0.66)^4^	0.47(0.26, 0.84)^2^	0.548	0.912
**Hydroxychloroquine**							
None	5298	1117	1.67e-05	ref	ref	ref	ref
≤0.5	223	37		0.77(0.56, 1.07)	1.06(0.76, 1.49)	0.2214	0.304
>0.5	739	99		0.63(0.51, 0.77)^4^	1.02(0.79, 1.32)	0.00135	0.0141
**Methotrexate**							
None	5689	1186	2.77e-06	ref	ref	ref	ref
≤0.5	195	24		0.56(0.38, 0.84)^3^	0.93(0.60, 1.45)	0.661	0.0499
>0.5	376	43		0.52(0.38, 0.71)^4^	1.05(0.72, 1.54)	0.154	0.12
**Etanercept**							
None	6204	1251	0.0021	ref	ref	ref	ref
>0	56	2		0.15(0.04, 0.61)^3^	0.29(0.07, 1.18)^1^	0.241	0.437
**Sulfasalazine**							
None	5719	1192	9.14e-08	ref	ref	ref	ref
>0	541	61		0.50(0.39, 0.65)^4^	0.82(0.59, 1.14)	0.107	0.637

^a^ adjusted for gender, age, Charlson comorbidity index, hypertension, hyperlipidemia, and all drugs except itself.

^1^p<0.1

^2^p<0.05

^3^p<0.01

^4^p<0.001.

As we hypothesize that antirheumatic drugs might have different effect on occurrence of CAD according to follow-up time. The Schoenfeld residual plot was used to investigate hypothesis. The Schoenfeld residual plots were used to visualize the relationship between time and drug effects on CAD. A Chi-squared test usually accompanies a residual plot to test for non-proportionality of hazards in the Cox model for each specific drug dose as we assign the nonusers as the reference group. This test is similar to the standard test for assessing the correlation between 'follow up time' and 'scaled Schoenfeld residuals' which are calculated separately for each drug level.

**Fig 2:**
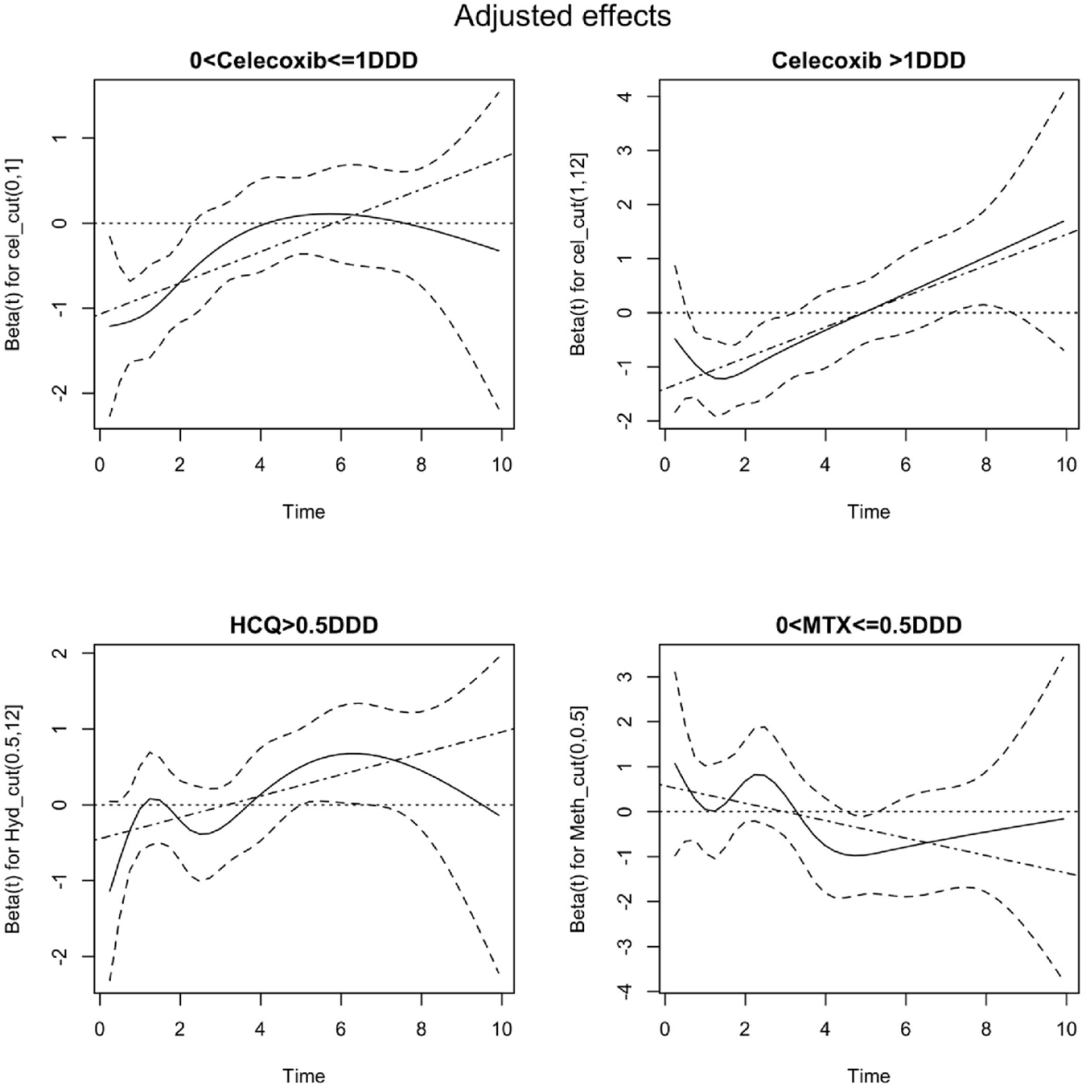
Smoothed Schoenfeld residual plots and fitted time-varying drug effect with confidence band when adjusted for covariates.

Fig 2 showed Smoothed Schoenfeld residual plots and fitted time-varying drug effect with confidence band when adjusted for covariates. Table 5 showed single drug effect of CAD in the RA study cohort. We found that time-varying drug effect happened at celecoxib. For low-dose celecoxib (DDD≦1) user, the crude and adjusted HR((95% CI) of CAD were 0.62(0.45, 0.85) and 0.47(0.34, 0.65) during the 4 year follow-up time; however, after 4 year, the crude and adjusted HR((95% CI) of CAD became 1.19(0.87, 1.64) and 0.98(0.70, 1.37). For high-dose celecoxib (DDD > 1) user, the adjusted HR of CAD during the period 0–4 years after diagnosis was 0.37(0.24, 0.58) (95% CI: 0.24–0.58) and during the 4–10 years period it was 1.29(0.85, 1.95) (95% CI: 0.85–1.95). The Wald z test for testing the effect difference between high and low- dose usage of Celecoxcib is 0.8574 with p-value = 0.39 showing that there is no significant difference in risk of CAD for high or low-dose Celecoxcib users during the first 4 years of follow-up. This result is also in concordance with that of the overlapping intervals shown in Table 5.

**Table 5:** Time-varying single drug effect on CAD in the RA study cohort.

Unadjusted Drug	RA duration 0~4 y	4~10y
**Celecoxib**		
≦1 DDD	0.62 (0.45–0.85)^3^	1.19 (0.87–1.64)
>1 DDD	0.35 (0.23–0.52)^2^	0.94 (0.67–1.20)
**HCQ**		
>0.5 DDD	0.50 (0.37–0.66)^4^	0.89 (0.65–1.20)
**Adjusted**		
**Celecoxib**		
≦1 DDD	0.47 (0.34–0.65)^4^	0.98 (0.70–1.37)
>1 DDD	0.37 (0.24–0.58)^4^	1.29 (0.85–1.95)
**HCQ**		
>0.5 DDD	0.79 (0.56–1.12)	1.45 (0.99–2.14)^1^
**MTX**		
≤0.5 DDD	1.38 (0.82–2.35)	0.45 (0.20–1.00)^1^

MTX, methotrexate; HCQ, hydroxychloroquine.

^1^p<0.1

^2^p<0.05

^3^p<0.01

^4^p<0.001.

## Discussion

In this nationwide population-based study of the impact of DMARDs on the potential risk for CAD following RA, we observed that a decreased CAD risk in the RA patients taking 2 different kinds of COX-2i in comparison with the nonusers. However, this phenomenon changed after about 4 years. Our study might be the first population-based cohort study design to report such an association. RA patients with both an average daily dose > 1 DDD or ≦ 1 DDD of celecoxib had reduced the risk of CAD than celecoxib nonusers. During the 0–4 year follow-up time, the adjusted HR(95% CI) of CAD were 0.47(0.34, 0.65) and 0.37(0.24, 0.58) for low-dose celecoxib (DDD≦1)) and high-dose celecoxib. The hazard ratios are quite overlapping and this does not reach significantly statistical difference. However, the etoricoxib users did not exhibit such a time-varying drug effect on decreasing CAD events. Various cyclooxygenase-2 selective inhibitors may affect the cardiovascular risk differently.

Our present study was unique for several reasons. First, to the best of our knowledge, current study may be the first nationwide study evaluating several types of antirheumatic drug use and the risk of CAD events in Asian RA population. Most previous studies were case-control designs or were carried out in the western countries. In addition, our study uses DDD to analyze the dosage of different antirheumatic drugs in the development of CAD in the RA cohort. This method of reporting COX-2i usage is more accurate and quantitative than just reporting whether there was or no usage. Moreover, we used Scaled Schoenfeld residuals to demonstrate the estimated preventive effect on CAD over time for drug usage. Furthermore, our cases included only one single race without the confounding factor of ethnicity and all our patients were followed up comprehensively with all kinds of medical services.

The use of some, but not all, NSAIDs displayed an association with an increased risk of CVD in the general population, particularly among the patients with established CAD[37]. Whether these drugs similarly increase risk of CVD in the RA patients, where their anti-inflammatory effects might reduce the risk, remain uncertain. In the general populations study, the VIGOR study showed that rofecoxib (Vioxx) was associated with an increased risk of acute myocardial infarction[24], and was removed from the market in 2004. The regrettable experience with rofecoxib has raised much awareness about the cardiovascular risk of other COX-2i or NSAIDs[15,26–28]. Because NSAIDs are widely used, it’s important to be aware of the downsides of taking an NSAID and to take steps to limit the risk. Therefore, FDA has strengthened its warning that NSAIDs increase risk of heart attack and stroke [38]. How can this apply to COX-2i and rheumatic diseases? Since these trials enrolled patients with colon adenoma, not a rheumatic diseases such as RA, it was not sure whether their results could also be generalized to patients with rheumatic diseases with chronic inflammation which can promote endothelial cell activation and vascular dysfunction. It may be the case that the first studies to identify CV risk were in this colon adenoma, but the key difference between these trials and the earlier arthritis trials was duration of intervention. A recently published small scale prospective cohort study of adults of either sex with arthritis who were initially diagnosed and taking COX-2i for < 3 months were included. Patients were grouped into nonselective and selective COX-2i groups with reference to treatment they received. Study clearly revealed that all NSAIDs exhibit variable CV risk; however, selective COX-2i found to exhibit more CV risk[37]. However, this study had small a sample size and a short duration of follow up and the analysis outcome only included demography of CV risk factors. Another PharMetrics claims database study on 107,908 RA patients (observed between 1999 and 2003) showed that risk of acute myocardial infarction was not related to COX-2i (rate ratio 1.11, 95% confidence interval 0.87–1.43) and biologic agents((RR 1.30, 95% CI 0.92–1.83), but significantly decreased with the current use of methotrexate[20]. In addition, follow up period was only 4 years[20]. Another case control study measured by self-reported medication use assessed through telephone interviews showed that celecoxib was more negatively associated with occurrence of non-fatal myocardial infarction compared with the NSAIDs non-users or the rofecoxib users[39]. However, the possibility of recall bias and uncontrolled confounding factors in this observational study limit the ability to make definitive conclusions. The association of celecoxib with a lower odds ratio of MI could have occurred by chance[39]. A more recently published large scale randomized controlled trials PRECISION (Prospective Evaluation of Celecoxib Integrated Safety versus Ibuprofen or Naproxen) evaluated the composite outcomes of cardiovascular death, nonfatal myocardial infarction and nonfatal stroke in 24,081 patients receiving celecoxib, ibuprofen or naproxen[40]. All the participants were victims of osteoarthritis or rheumatoid arthritis who required daily NSAIDs treatment and were at increased cardiovascular risk. The results of the trial show that at moderate doses, celecoxib was found to be noninferior to ibuprofen or naproxen with regard to cardiovascular safety at for a mean treatment duration of 20.3±16.0 months and a mean follow-up period of 34.1±13.4 months. Several concerns related to this trial were noted with the interpretation of PRECISION trial. First, PRECISION is questioned that it is not a study of arthritis patients at high cardiovascular risk. It mostly included osteoarthritis patients at low cardiovascular risk–cardiac event rates were roughly 1% per year[41]. Secondly, the validity of the conclusions around non‐inferiority was criticized due to low power, low drug compliance (68.8% of the patients stopped taking the study drug), and high rate of lost to follow-up (27.4% of the patients discontinued follow-up) [41].Our present study is unique in that it involves a cohort study design with minimal risk of recall bias and had longer duration of follow-up period. These conflicting data may be attributed to selection or recall bias and variations in sample size, different outcome measures such as the CAD criteria, the patients’ race, and the study design.

This study is the first to report an association of decreased CAD risk in RA patients taking 2 different kinds of COX-2i in comparison with the nonusers. What is “the possible mechanism of the beneficial effects of COX-2 therapy in CAD prevention?” CAD is a severe atherosclerosis disease affecting the coronary artery. One of the early stage of atherosclerosis change is endothelial dysfunction[42,43]. Accumulating evidences have suggested that endothelial dysfunction may predict a clinical outcome in the CAD patients [44,45]. Indeed, several studies suggested that celecoxib improved endothelial function and also had potentially beneficial effects on coronary artery blood flow[46,47].

Recent studies suggest that DMARDs use, such as methotrexate, sulfasalazine, leflunomide, is associated with a reduced risk of CAD in RA patients[18–20]. Two observational studies showed anti-TNF agents were associated the decreased risk of new cardiovascular events[22–23]. A very recently published study revealed use of hydroxychloroquine was associated with a 72% reduction in the risk of incident CVD in a retrospective inception cohort of RA patients. However, it was not seen in our study. There are no significant protective effect for CAD among users of sulfasalazine, methotrexate, hydroxychloroquine, or etanercept after adjusting for demographic characteristics, medical comorbidities and other antirheumatic drug use. We speculated the possibility of not enough power to detect a difference in the etanercept group(adjusted HR of CAD was 0.31 with 95% CI, 0.08 to 1.27). Lastly, we guess could there be drug interactions among different kinds of antirheumatic drug affecting CVD outcomes thus confounding the results?

### Strengths and limitations

The foremost advantage of this study is the use of a nationwide, population-based claim database, which can minimize selection bias and recall bias. Previous studies either used registra data or hospital used data. Another possible strength is that our study included only one single race without the confounding factor of ethnicity. Third, due to 99.6% coverage of insurance population in Taiwan, all our patients were followed up comprehensively with all kinds of medical services. However, there are also some limitations deserving attention. First, no information was provided on disease activity, such as clinical activity index (such as rheumatoid factor, radiographic data) and no information was available on the degree of systemic inflammation (such as C-reactive protein, erythrocyte sedimentation rate) which may have an impact on the risk of CAD. These were not available in the current administrative databases. Another major limitation of the study was the lack of body mass index and lifestyle habits of the patients (such as smoking, physical activities) which may influence the risk of CAD. Thus, we could not conduct sophisticated tests adjusting for these variables. Third, potential confounding by indications of drugs prescription in this observational study should be taken into consideration. It is likely subjects considered to have a lower CV risk were prescribed celecoxib and etoricoxib more frequently than subject with high risk. COX-2i may have been avoided in patients perceived by their physician to be at higher risk of CV events and this may be a source of bias. Fourth, though we have adjusted for some confounders such as hypertension, hyperlipidemia, diabetes, stroke history, peripheral artery disease, chronic renal failure and COPD, there are still some other variables which were not taken in account such as family history of CAD, use of aspirin, statin, antihypertensives and corticosteroids. Bias due to these factors might still remain. Additionally, the diagnosis of RA, CAD, and medical comorbidities were based solely on the ICD codes from the NHI claim database, instead of a medical chart review. As Thomas SL et al [48] found in his validation study of RA database diagnosis this definition has 80% sensibility and 80% specificity, so some misclassification is likely to exist and this must be acknowledged. However, the BNHI routinely audits the accuracy of diagnoses by randomly sampling patient charts. This BNHI audit has enhanced coding accuracy[49]. Therefore, the NHI claim database is an established research database and there have been some independent studies demonstrating the validity of the NHIRD data[50,51].

### Conclusion

In this 10-year population-based cohort study, we found that decreased CAD risk in RA patients taking 2 different kinds of COX-2i in comparison with nonusers. Use of celecoxib was associated with reduced CAD risks among RA patients during the 4 year follow-up time; however, this phenomenon was changed overtime, after about 4 years. The effect of etoricoxib with reduced CAD risks among RA patients remained constant over the follow up time.
